# Acute post‐traumatic dermoid peritonitis: A rare entity

**DOI:** 10.1002/ccr3.6477

**Published:** 2022-10-17

**Authors:** Sami Fendri, Haitham Rejab, Ayman Trigui, Bassem Abid, Youssef Majdoub, Ahmed Bouzid, Kais Fourati, Salah Boujelbene

**Affiliations:** ^1^ General Surgery Department Habib Bourguiba Hospital Sfax Tunisia; ^2^ Sfax Medical School University of Sfax Sfax Tunisia; ^3^ Departement of Anatomy Sfax Medical School Sfax Tunisia

**Keywords:** dermoid, ovarian cyst, peritonitis

## Abstract

Rupture of ovarian dermoid‐cyst is rare case. We report the case of a woman admitted for acute post‐traumatic abdominal pain due to ruptured ovarian cyst. The patient was operated and we found a very abundant peritoneal effusion with left ovarian cyst which was broken. we performed a left adnexectomy.

## Abstract 2

Rupture of an ovarian teratoma with dermoid peritonitis is a diagnosis that we should think of in case of an acute peritonitis in a women whatever her age is. It is a rare diagnosis especially when have eliminate other diagnosis such as appendicitis, cholecystits, or bowel perforation.

## 1 INTRODUCTION

Dermoid peritonitis is secondary to intraperitoneal rupture of a dermoid cyst (or mature teratoma). This is the most common complication after torsion. The rupture can be in another organ or in the peritoneal cavity.

## 2 OBSERVATION

A 75‐year‐old patient, with no particular pathological history, admitted for acute post‐traumatic abdominal pain. Indeed, she was the victim of a domestic accident: She was fallen from her height.

The clinical examination found the temperature at 38°C, she has a stable hemodynamic state with diffuse abdominal tenderness and maximum pain at the pelvic level and significant abdominal distension. She has not an abdominal impact point.

Biology showed a biological inflammatory syndrome with hyperleukocytosis at 16,500 elements/mm^3^ with a CRP at 78 mg/L.

CT scan found a left ovarian cyst with a dermoid appearance (calcifications with heterogeneous contents) associated with abundant intraperitoneal effusion with diffuse infiltration of intra‐abdominal fat. A ruptured ovarian cyst in the peritoneum was concluded.

**FIGURE 1:**
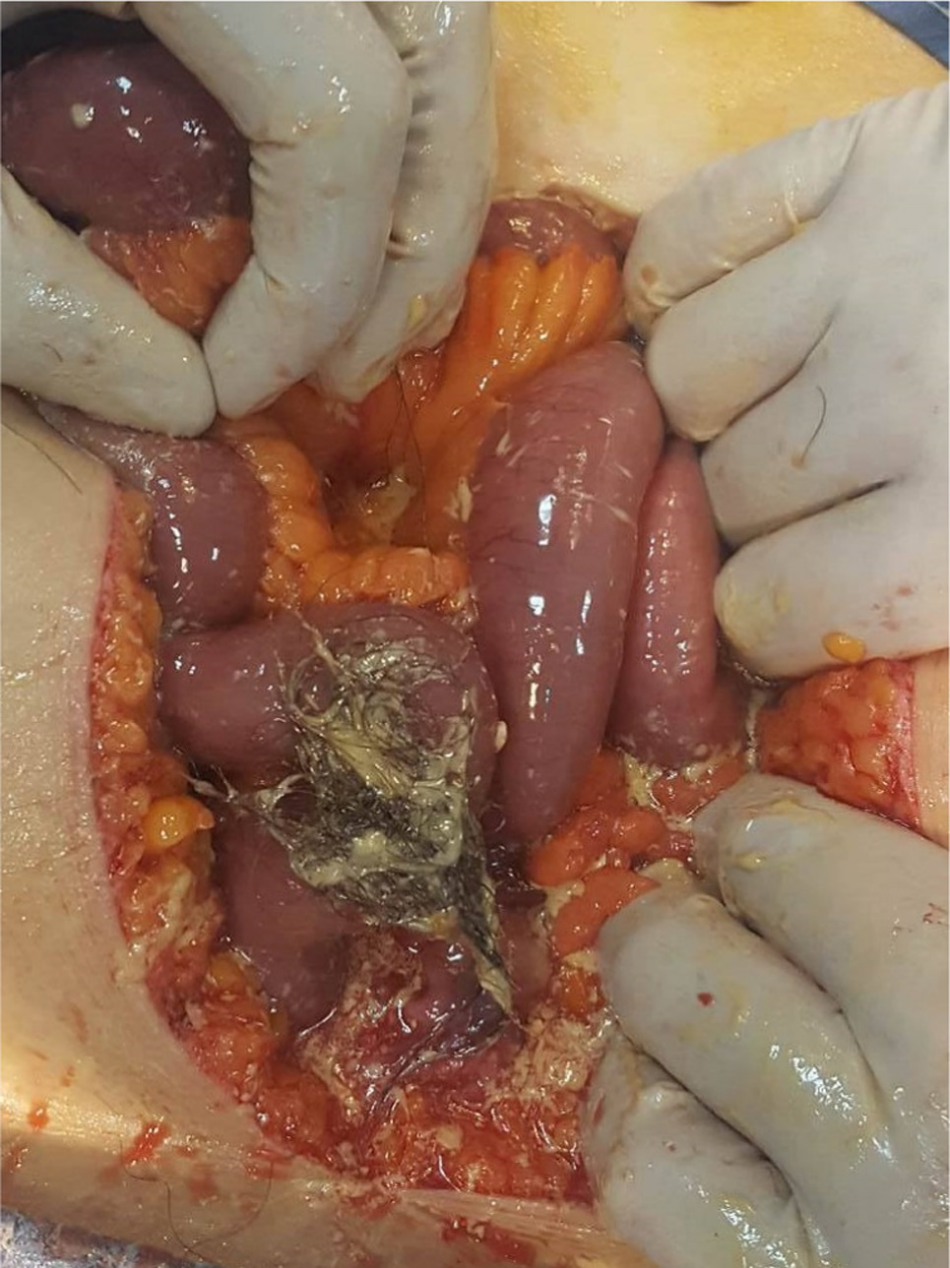
Operative view: Peritoneal effusion with sebum, tufts of hair and some hard calcium debris.

**FIGURE 2:**
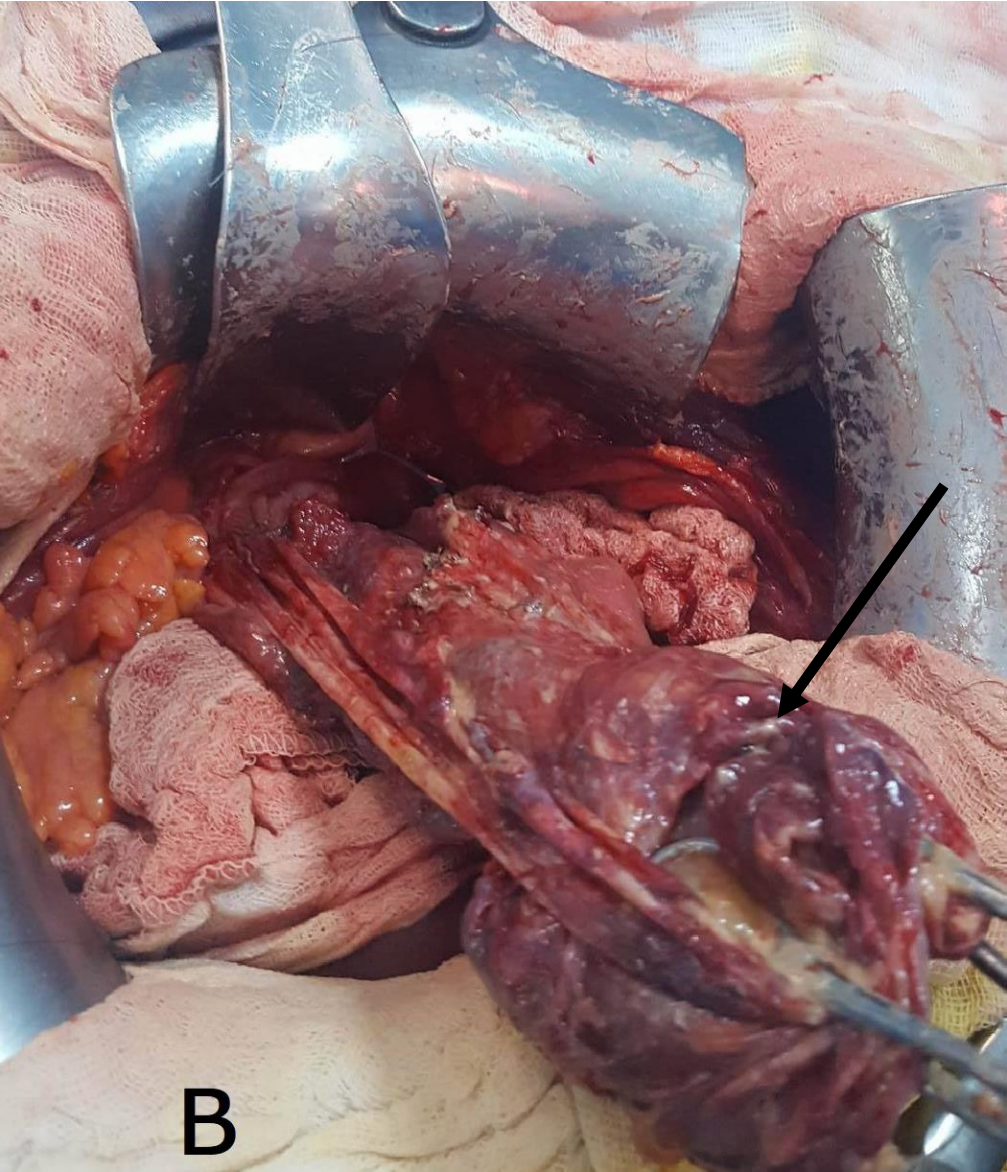
Left ruptured ovarian cyst (black arrow).

**FIGURE 3:**
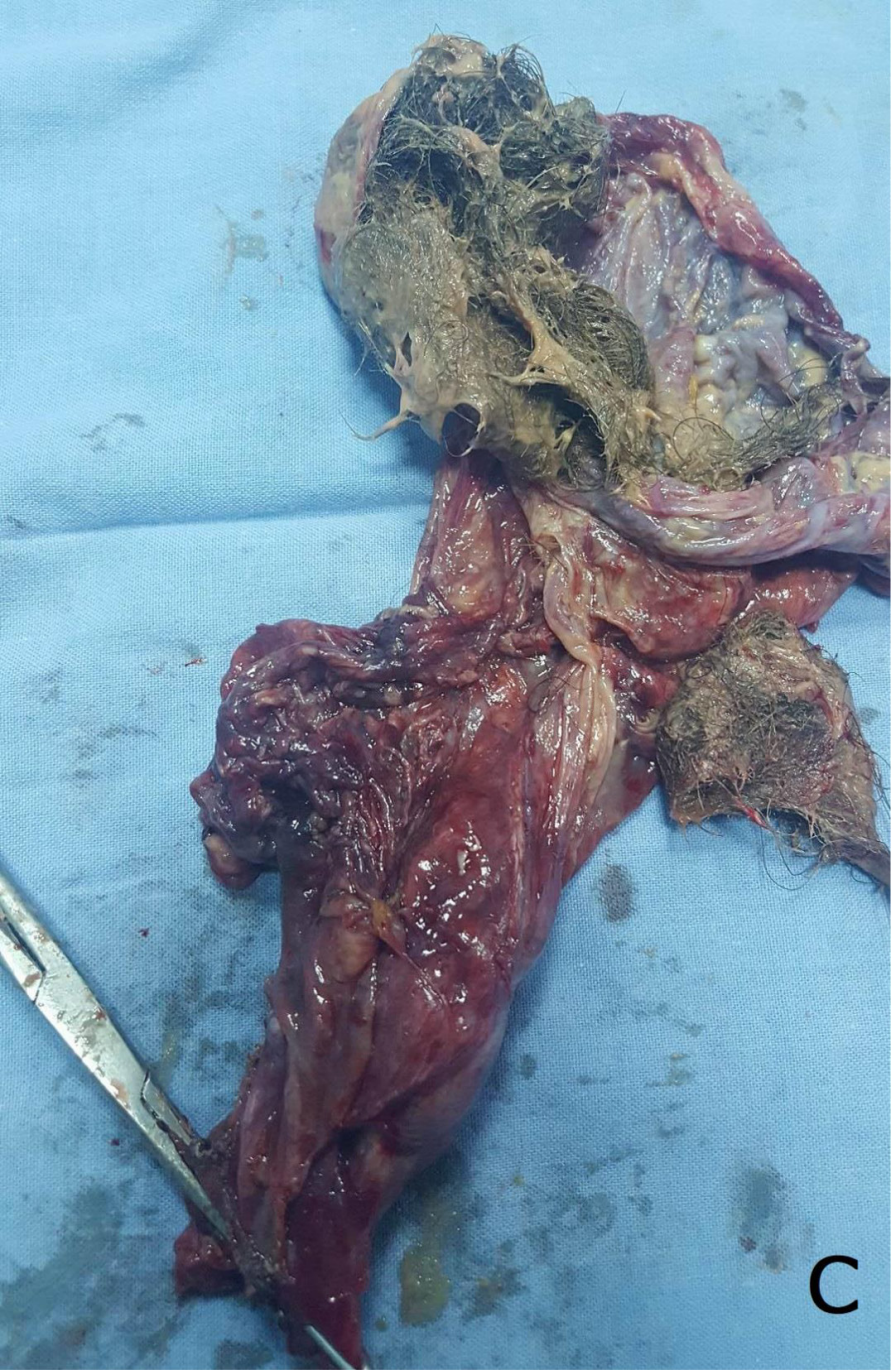
Specimen of left adnexectomy

The patient was operated urgently. We found a very abundant peritoneal effusion made of sero‐hematic fluid, sebum, tufts of hair, and some hard calcium debris (Figure 1). There was also a left ovarian cyst which was broken (Figure 2). Regarding her age, we performed a left adnexectomy removing the ruptured dermoid cyst with abundant peritoneal cleaning (Figure 3).

The postoperative course was simple after 2 days in intensive care unit.

## 3 DISCUSSION

The dermoid cyst is a very common ovarian tumor (approximately 25% of ovarian tumors).^1^ It occurs mainly in women during period of genital activity.^2^

In half of the cases, it is asymptomatic.^2^, ^3^, ^4^, ^5^ It can be discovered as a result of pelvic pain, menstrual irregularities, or complications.^2^, ^5^

Diagnosis is based on imaging data, particularly ultrasound and CT, which highlight the presence of heterogeneous calcium and fatty components.^6^ The complications of these ovarian cysts are dominated by torsion (15%), rupture (1.3%), infection (1%–2%), and more rarely hemorrhage.^2^

Rupture in the peritoneum is complicated by chemical peritonitis.^2^, ^7^, ^8^ The treatment of ruptured dermoid cysts of the ovary is surgical, either by laparotomy or by laparoscopy.

Cystectomy is the standard procedure in young women, while oophorectomy or adnexectomy is most often performed in postmenopausal women.^9^, ^10^

Abundant washing of the peritoneal cavity should be performed with saline solution and all visible particles of dermoid material (hair, bone, fat, and sebum) should be removed.^10^

Examination of the anatomo‐pathological specimen is essential and mandatory in order to confirm the diagnosis and to look for an immature dermoid component, in which case further surgery or chemotherapy should be discussed.

Rubod and then El Moussaoui have reported a similar case treated by adnexectomy as we have done to our patient.^4^, ^9^

## 4 CONCLUSION

Dermoid peritonitis is a rare condition secondary to sudden rupture (acute form) or cracking (torpid form) of an ovarian teratoma. This diagnosis should be considered in case of peritonitis with nonexplained etiology, especially when the CT scan reveals a mature teratoma. It is important to evoke the acute form in a woman of childbearing age, presenting with severe pelviperitonitis.

## AUTHOR CONTRIBUTIONS

S Fendri conceived the idea for the document and contributed to the writing and editing of the manuscript. H Rejab contributed to the writing and editing of the manuscript. A Trigui, B Abid, Y Majdoub, A Bouzid, and K Fourati reviewed and edited the manuscript. S Boujelbene contributed to the literature review, manuscript writing, editing, and review of the manuscript. All authors read and approved the final manuscript.

## FUNDING INFORMATION

No fundings needed for this publication.

## CONFLICT OF INTEREST

None for all authors.

## ETHICAL APPROVAL

Personal data have been respected.

## CONSENT

Written informed consent was obtained from the patient to publish this report in accordance with the journal's patient consent policy.

## Data Availability

Personal data of the patient were respected.
